# Editorial: Safety of drugs and CAM products in pregnancy and breastfeeding: evidence from clinical toxicology

**DOI:** 10.3389/fphar.2023.1340283

**Published:** 2023-12-06

**Authors:** Giada Crescioli, Niccolò Lombardi, Alfredo Vannacci

**Affiliations:** ^1^ Department of Neurosciences, Psychology, Drug Research and Child Health, Section of Pharmacology and Toxicology, University of Florence, Florence, Italy; ^2^ Tuscan Regional Centre of Pharmacovigilance, Florence, Italy; ^3^ Joint Laboratory of Technological Solutions for Clinical Pharmacology, Pharmacovigilance and Bioinformatics, University of Florence, Florence, Italy

**Keywords:** drug, complementary and alternative medicine, safety, efficacy, clinical toxicology, pregnancy, breastfeeding

Perinatal medicine and pharmacology focus on the wellbeing and health of women and newborns throughout all stages of pregnancy, from conception to birth, and in the first year of extra uterine life (World Health Organization -WHO, 2023). The role of research in clinical pharmacology, toxicology, pharmacovigilance, and pharmacoepidemiology is crucial in understanding the risk/benefit balance of drugs and products belonging to complementary and alternative medicine (CAM) during pregnancy and breastfeeding. In this context, the role of clinical pharmacologists is of great importance, as their knowledge can be utilised to optimise dosage and treatment regimens and to assess the relationship between exposure to active compounds and clinical outcomes, both in terms of efficacy, and safety, such as adverse drug reactions (ADRs) (Crescioli et al., 2022).

Mothers are commonly exposed to drugs during pregnancy, at the time of birth, or in the postpartum period, with drug therapy exposure increasing in recent years (Lupattelli et al., 2014; Bettiol et al., 2018; Donald et al., 2020). However, perinatal use of drugs and CAM products is not always supported by definitive scientific evidence. In fact, along with children and elderly subjects, pregnant women are still considered one of the so-called “therapeutic orphans” because most drugs have never been studied in this population during their development phase (Ayad and Costantine, 2015). The evidence is even more limited in the case of CAM products (Bettiol et al., 2018; Crescioli et al., 2023). According to the American College of Obstetricians and Gynecologists, even in the case of pregnancy, the patient’s informed consent should be sufficient to allow inclusion in the study (ACOG, 2007). However, the possibility of causing teratogenic effects, fetotoxicity or damage to newborns has always limited the participation of pregnant and breastfeeding women, and often also of women of childbearing age, in randomised clinical trials (RCTs) (Niebyl and Simpson, 2008). Most drugs prescribed to pregnant and breastfeeding women are therefore used *off-label*, and over 90% do not provide appropriate information for this kind of patients within the summary of product characteristics (Ren et al., 2021).

Various strategies, including the allocation of specific funds by regulatory agencies, the recruitment of researchers with diverse areas of expertise, and the revision of legislation related to RCTs (Biggio, 2020), could represent a turning point in increasing knowledge and, consequently, the quality of care for pregnant women (Byrne et al., 2020; Caritis and Venkataramanan, 2021). Specific studies on women undergoing therapy during pregnancy and/or breastfeeding would be useful not only to determine the effectiveness and safety of such therapies in this subgroup, but also to establish care and monitoring protocols to ensure successful treatment outcomes in clinical practice. In fact, without specific data on pregnant and breastfeeding women before introducing a drug or a CAM product to the market, post-marketing surveillance becomes crucial to assess not only the safety of drugs and possible adverse events, but also their impact on the child’s growth both in the medium and long term (Illamola et al., 2018).

Similarly, the use of so-called *big data* from computerised health data management systems could provide important information about real clinical practice in this population (Schneeweiss, 2014).

The purpose of this Research Topic is to bring together a Research Topic of new original research, systematic reviews, meta-analysis, case reports and observational studies that could provide new data supporting the use of drugs, CAM products, and even traditional Chinese medicine during pregnancy and breastfeeding. Xie et al. performed a systematic review of literature and meta-analysis on the efficacy and safety of Chinese herbal medicine (CHM) for threatened miscarriage. According to their results, the analysis of 57 RCTs (5,881 patients) showed that compared with western medicine (WM) alone, CHM alone showed significant higher incidence of continuation of pregnancy after 28 gestational weeks, and continuation of pregnancy after treatment, as well as higher levels of β-human chorionic gonadotropins (β-hCG). Similar results were observed for all these outcomes comparing WM alone to the combination of CHM with WM. Another systematic review and meta-analysis performed by Chen et al., showed that Gushen Antai pills combined with dydrogesterone alone can reduce the incidence of early pregnancy loss and alleviate clinical symptoms. Also, this analysis demonstrated that integrating Gushen Antai Pills and dydrogesterone is more effective than using dydrogesterone alone in improving hormone levels (serum levels of progesterone, β-HCG and estradiol) for women with threatened miscarriage. The risks associated with acetaminophen use in inducing reproductive and neurobehavioral dysfunctions or hepatotoxicity in offspring was clarified by Wu et al.. In this mini review, authors concluded that exposure to acetaminophen during pregnancy to treat pain or other symptoms may disrupt endocrine functions, including brain and liver tissues, involving several intracellular and extracellular mechanisms such as acetaminophen-induced cytotoxicity, metabolism, mitochondrial oxidative stress, DNA damage and microcirculatory dysfunction. Another systematic review and meta-analysis evaluated the risk of atopic dermatitis in children born to mothers exposed to antibiotics (Wan and Yang). Prospective, retrospective, cross-sectional, and case-control studies published between 2002 and 2022 were included in the analysis. Results suggested that maternal exposure to antibiotics may lead to a modestly increased risk of atopic dermatitis in offspring. This study underlines also the high interstudy heterogeneity and bias in exposure and outcome assessment that deeply limit the evidence. Observational studies and qualitative research could delve into the use of CAM products and drugs in this population. Liu et al. observed that, among 2803 women recruited from a comprehensive obstetric care centre in Shanghai, the prevalence of dietary supplement use during pregnancy (all trimesters) and in the first, second, and third trimester was 94.8%, 96.2%, 93.8%, and 94.4%, respectively. Yeung et al. found that healthcare providers who use resources to advise on medication use during breastfeeding have an improved understanding of current practice approaches. In particular, upper area under the curve ratio (UAR) would confer multiple benefits over existing resources and future research could improve an optimal uptake of the UAR to ameliorate healthcare providers' advising practices during breastfeeding.

*Ex vivo* and *in vitro* studies could verify the transplacental passage of commonly used active compounds. Since Saint John’s wort and valerian are widely used for mental health, and safe medications for mild mental diseases in pregnancy are still lacking, Spiess et al. evaluated the transplacental transport of hyperforin, hypericin, and valerenic acid. Perfusion data showed that small amounts of hyperforin passed into the foetal circuit, while hypericin did not cross the placental barrier. Valerenic acid, on the other hand, equilibrated between the maternal and foetal compartments.

Anecdotal case reports may be useful to raise safety signals. Li et al., reported a rare case of placenta interception observed during caesarean section caused by carbetocin. Carbetocin was injected intravenously after delivery to prevent postpartum haemorrhage.

In conclusion, clinical and observational research could promote a personalised approach in pharmacology during pregnancy and breastfeeding. The personalised approach in clinical pharmacology offers the opportunity to design preventive and therapeutic interventions that leverage the integration of a variety of observations, not exclusively from a single one, referring to a particular pregnancy through the construction of mathematical algorithms (Stevenson et al., 2021). No disease will then be classified exclusively in terms of biological or social determinants to understand its aetiology, even when determinants include a set of biological and environmental factors. The application of innovative computational techniques is revolutionising the practice of medicine and allows for addressing each case with a multidisciplinary approach (Gracie et al., 2011).

## References

[B1] ACOG (2007). ACOG committee opinion No. 377: research involving women. Obstet. Gynecol. 110, 731–736. 10.1097/01.AOG.0000263926.75016.db 17766625

[B2] Ayad M. Costantine M. M. (2015). Epidemiology of medications use in pregnancy. Semin. Perinatol. 39, 508–511. 10.1053/j.semperi.2015.08.002 26358804 PMC4628584

[B3] Bettiol A. Lombardi N. Marconi E. Crescioli G. Bonaiuti R. Maggini V. (2018). The use of complementary and alternative medicines during breastfeeding: results from the Herbal supplements in Breastfeeding InvesTigation (HaBIT) study. Br. J. Clin. Pharmacol. 84, 2040–2047. 10.1111/bcp.13639 29768673 PMC6089807

[B4] Biggio J. R. J. (2020). Research in pregnant subjects: increasingly important, but challenging. Ochsner J. 20, 39–43. 10.31486/toj.19.0077 32284681 PMC7122255

[B5] Byrne J. J. Saucedo A. M. Spong C. Y. (2020). Task force on research specific to pregnant and lactating women. Semin. Perinatol. 44, 151226. 10.1016/j.semperi.2020.151226 32173086

[B6] Caritis S. N. Venkataramanan R. (2021). Obstetrical, fetal, and lactation pharmacology-a crisis that can no longer be ignored. Am. J. Obstet. Gynecol. 225, 10–20. 10.1016/j.ajog.2021.02.002 34215351

[B7] Crescioli G. Bonaiuti R. Corradetti R. Mannaioni G. Vannacci A. Lombardi N. (2022). Pharmacovigilance and pharmacoepidemiology as a guarantee of patient safety: the role of the clinical pharmacologist. J. Clin. Med. 11, 3552. 10.3390/jcm11123552 35743619 PMC9225198

[B8] Crescioli G. Maggini V. Raschi E. Gonella L. A. Luxi N. Ippoliti I. (2023). Suspected adverse reactions to medications and food supplements containing Serenoa repens: a worldwide analysis of pharmacovigilance and phytovigilance spontaneous reports. Phytother. Res. 37, 5289–5299. 10.1002/ptr.7960 37463655

[B9] Donald S. Sharples K. Barson D. Horsburgh S. Parkin L. (2020). Patterns of prescription medicine dispensing before and during pregnancy in New Zealand, 2005-2015. PLoS One 15, e0234153. 10.1371/journal.pone.0234153 32484824 PMC7266349

[B10] Gracie S. Pennell C. Ekman-Ordeberg G. Lye S. McManaman J. Williams S. (2011). An integrated systems biology approach to the study of preterm birth using “-omic” technology--a guideline for research. BMC Pregnancy Childbirth 11, 71. 10.1186/1471-2393-11-71 21992798 PMC3205030

[B11] Illamola S. M. Bucci-Rechtweg C. Costantine M. M. Tsilou E. Sherwin C. M. Zajicek A. (2018). Inclusion of pregnant and breastfeeding women in research - efforts and initiatives. Br. J. Clin. Pharmacol. 84, 215–222. 10.1111/bcp.13438 28925019 PMC5777434

[B12] Lupattelli A. Spigset O. Twigg M. J. Zagorodnikova K. Mårdby A. C. Moretti M. E. (2014). Medication use in pregnancy: a cross-sectional, multinational web-based study. BMJ Open 4, e004365. 10.1136/bmjopen-2013-004365 PMC3927801 24534260

[B13] Niebyl J. Simpson J. (2008). Teratology and drugs in pregnancy. Glob. Libr. women’s Med. 10.3843/GLOWM.10096

[B14] Ren Z. Bremer A. A. Pawlyk A. C. (2021). Drug development research in pregnant and lactating women. Am. J. Obstet. Gynecol. 225, 33–42. 10.1016/j.ajog.2021.04.227 33887238

[B15] Schneeweiss S. (2014). Learning from big health care data. N. Engl. J. Med. 370, 2161–2163. 10.1056/NEJMp1401111 24897079

[B16] Stevenson D. K. Wong R. J. Aghaeepour N. Maric I. Angst M. S. Contrepois K. (2021). Towards personalized medicine in maternal and child health: integrating biologic and social determinants. Pediatr. Res. 89, 252–258. 10.1038/s41390-020-0981-8 32454518 PMC8061757

[B17] World Health Organization -WHO (2023). Maternal health. Available at: https://www.who.int/health-topics/maternal-health#tab=tab_1.

